# An Open-Label, Comparative, Parallel Clinical Trial of the Safety, Pharmacokinetics and Immunogenicity of the Ustekinumab Biosimilar GNR-068 and the Originally Developed Ustekinumab as a Reference Drug After a Single Subcutaneous Administration in Healthy Male Volunteers

**DOI:** 10.3390/antib15040077

**Published:** 2026-08-12

**Authors:** Ivan Lyagoskin, Alena Agafonova, Ivan Shevchenko, Nina Akhtyamova-Givirovskaya, Oksana Markova, Marina Pantyushenko, Rakhim Shukurov, Ravil Khamitov

**Affiliations:** JSC “GENERIUM”, 14, Vladimirskaya Street, Volginskiy 601125, Vladimir Region, Russia; aaagafonova@generium.ru (A.A.); iv.shevchenko@generium.ru (I.S.); neakhtyamova-givirovskaya@generium.ru (N.A.-G.); oamarkova@generium.ru (O.M.); pantushenko@ibcgenerium.ru (M.P.); shukurov@ibcgenerium.ru (R.S.); khamitov@ibcgenerium.ru (R.K.)

**Keywords:** biosimilar, pharmacokinetics, immunogenicity, ustekinumab, GNR-068, Stelara^®^, clinical trial

## Abstract

GNR-068 is a proposed biosimilar to the ustekinumab reference product (RP), which works through the antagonism of interleukin 12 and interleukin 23. Ustekinumab RP is used for the treatment of chronic inflammatory conditions, including certain forms of plaque psoriasis, psoriatic arthritis, Crohn’s disease, and ulcerative colitis. **Objectives**: The purpose of the study is to study the safety, pharmacokinetics, and immunogenicity of the ustekinumab biosimilar GNR-068 and the reference drug Stelara^®^ after a single subcutaneous administration in healthy male volunteers. **Methods**: This was an open-label, randomized, comparative, parallel-group clinical trial of the safety, pharmacokinetics (PK), and immunogenicity of GNR-068 (JSC GENERIUM) and Stelara^®^ (Manufacturer: Silag AG, Switzerland; RU Holder: JOHNSON & JOHNSON) after a single subcutaneous administration of 45 mg in healthy volunteers. During the trial, 146 volunteers were screened, and 122 were randomized. **Results**: The results showed that PK similarity was established based on 90% confidence intervals (CIs) for the ratios of geometric means of the primary endpoints of area under the concentration-time curve from time 0 extrapolated to infinity (AUC_0–∞_) and maximum observed serum concentration (Cmax) being contained within the pre-specified margin of 80.00–125.00%. The incidence of total ADA was lower in the GNR-068 group compared with the reference product group. Adverse events were similar between treatment groups and consistent with the safety profile of the ustekinumab RP. **Conclusions**: These results indicate that GNR-068 and the ustekinumab RP share similar PK and safety profiles.

## 1. Introduction

Until recent years, the leading strategy in medicine remained the “treat to improvement” principle, which set partial resolution of the acute process and reduction in symptom severity as its primary goal. However, over the past 15–20 years, the evolving understanding of the pathogenesis of chronic diseases (particularly psoriasis) [1,2,3,4,5,6,7] and breakthroughs in the pharmaceutical industry have driven a global transition toward the “treat to target” (T2T) concept. This concept aims to achieve strict, measurable, and long-term therapeutic goals [8,9]. The introduction of highly effective biologic therapies into clinical practice has fundamentally altered the prognosis for patients suffering from dermatological, rheumatological, and inflammatory bowel diseases [3].

Ustekinumab, a fully human monoclonal antibody targeting the shared p40 subunit of interleukin 12 (IL-12) and interleukin 23 (IL-23), has become one of the landmark biologics in the treatment of systemic immune-mediated inflammatory diseases. Ustekinumab is approved for a wide spectrum of indications: moderate-to-severe plaque psoriasis, psoriatic arthritis, Crohn’s disease, and ulcerative colitis. According to published data, psoriasis affects 2–4% of the population in different countries [10,11,12,13,14], and it is often accompanied by a wide range of comorbid conditions, including motor disorders and endocrine and cardiovascular disorders, as well as inflammatory bowel diseases [3,15]. By blocking the p40 subunit, ustekinumab suppresses two regulatory pathways, namely the IL-12-dependent differentiation of Th1 cells and the IL-23-dependent signaling pathway of Th17 cells [16,17,18], both of which play a pivotal role in the pathogenesis of psoriasis and its comorbidities. The application of biologic therapy is directed toward achieving complete or almost complete skin clearance and maximizing the restoration of quality of life. At the same time, the accessibility of innovative treatment has come to the forefront. The introduction of biosimilars has become a crucial tool for expanding patient access to highly effective therapies, stimulating market competition, and significantly reducing the economic burden on healthcare systems [19].

The drug GNR-068 (JSC GENERIUM, Russia) proposed for trial is a biosimilar to Stelara^®^ (manufacturer: Cilag AG, Switzerland; RU holder: Johnson & Johnson). This trial was designed to compare the safety and pharmacokinetics of GNR-068 and Stelara^®^ to support the registration of GNR-068, a solution for subcutaneous administration, in the Russian Federation.

According to the Guidelines for the Expertise of Medicinal Products, the selected comparator medicinal product must be the original one registered in the Russian Federation with a complete dossier. The biosimilar’s active ingredient must be molecularly and biologically similar to that of the comparator. The route of administration, dosage, and dosage form must not differ [20].

Stelara^®^ meets all of the above conditions as the original (and only) ustekinumab-based product registered in the Russian Federation. Its active ingredient and excipients are identical in composition and content to those of GNR-068, as is the dosage form.

## 2. Materials and Methods

The bioequivalence between GNR-068 and a Stelara^®^ formulation was investigated in a clinical trial: a phase I trial to evaluate its safety and pharmacokinetics (UKM-PS-I, v2. 10 March 2022). The trial was conducted in accordance with the Declaration of Helsinki. All procedures and the informed consent form were reviewed and approved by an institutional review board. Informed consent was obtained from each participant.

### 2.1. Materials

GNR-068 (JCS GENERIUM, Volginskiy, Russia) is formulated with the same active ingredient: 45 mg (45 mg/0.5 mL) of ustekinumab. Excipients: sucrose—38 mg; L-histidine (including L-histidine hydrochloride monohydrate)—0.5 mg; polysorbate 80—0.02 mg; hydrochloric acid to adjust pH to 6.0; and water for injection—up to 0.5 mL, USP, as Stelara^®^ (Cilag AG, Schaffhausen, Switzerland).

Pharmacokinetic (PK) and anti-drug antibody (ADA) analyses were carried out using 96-well MaxiSorp plates (Cat# 456537, Nunc, Roskilde, Denmark). The recombinant p40 subunit of interleukins 12 and 23, used for the analysis of neutralizing antibodies (NAbs), was produced at JSC GENERIUM. Biotinylated ustekinumab (GNR-068) was prepared using the Biotin (Type A) Fast Conjugation Kit (Cat# ab201795, Abcam, Cambridge, UK). Human monoclonal antibodies specific to the ustekinumab binding site (Cat# HCA210, Bio-Rad, Neuried, Germany) and horseradish peroxidase (HRP)-conjugated streptavidin (Cat# ab7403, Abcam, Cambridge, UK) were also used in the ADA assessment methods.

Polyclonal antibodies raised in rats against Stelara^®^ were produced at JSC GENERIUM for use in PK analysis. Rat polyclonal antibodies conjugated to HRP were prepared using the LYNX Rapid HRP Antibody Conjugation Kit (Cat# LNK004P, Bio-Rad, Kidlington, UK).

Tetramethylbenzidine (TMB) (Cat# 01016-1) was purchased from Bektor Corporation (Aurora, CO, USA).

### 2.2. Phase I Trial

#### 2.2.1. Characteristics of the Participants

Eligible participants were healthy male volunteers, aged 18–45 years, weighing 60.0–90.0 kg (18.5–30.0 kg/m^2^ body mass index [BMI]), who provided written informed consent. Exclusion criteria included a history of major organ dysfunction, including but not limited to the liver, kidneys, and cardiovascular system; positive test results for hepatitis B or C, human immunodeficiency virus, or Treponema pallidum; current or past alcoholism; and other criteria according to the physician’s decision.

#### 2.2.2. Conducting the Trial in Accordance with Ethical Principles

The clinical trial was conducted in accordance with the UKM-PS-I clinical trial protocol; Good Clinical Practice (GCP) requirements; the World Medical Association Declaration of Helsinki; GOST R 52379-2005 “National Standard of the Russian Federation “Good Clinical Practice”; Federal Law No. 61-FZ of 12 April 2010 “On the Circulation of Medicines”; Federal Law No. 323-FZ of 21 November 2011 “On the Fundamentals of Health Protection of Citizens in the Russian Federation”; and Law of the Russian Federation No. 1499-I of 28 June 1991 “On Medical Insurance of Citizens in the Russian Federation”.

#### 2.2.3. Trial Design

The safety and pharmacokinetics of GNR-068 were compared with those of Stelara^®^ following a single 45 mg subcutaneous dose in healthy volunteers.

The objectives of the trial were to evaluate (1) the safety profile of GNR-068 compared with Stelara^®^, including the incidence, severity, and causality of adverse events (AEs) based on clinical and laboratory assessments; (2) the bioequivalence between GNR-068 and Stelara^®^ using the primary pharmacokinetic parameters AUC_0–t_; AUC_0–∞_, and Cmax; (3) the secondary pharmacokinetic parameters (AUC_0–t_; AUC_0–∞_, Cmax, Tmax, T1/2, CL, and Vd); and (4) the immunogenicity profile, including the frequency, titer, and neutralizing activity of ADA.

#### 2.2.4. Statistical Analysis

##### Sample Size Determination

The sample size was estimated using NCSS PASS v.15. Assuming a true test/reference ratio of 1.00 for a within-subject CV of 33% based on published data for ustekinumab [21], a two-sided α of 0.05, 80% power, and bioequivalence acceptance limits of 80.00–125.00% for the ratio of geometric means, at least 92 evaluable subjects were required. After adding 25% for potential dropouts, the final sample size was set to 122 subjects.

##### Randomization

Volunteers were allocated to treatment groups using stratified randomization in a 1:1 ratio between the trial drug (GNR-068) and the comparator drug (Stelara^®^). Volunteer body weight served as a stratification factor during randomization. Three strata were defined within each group: 60.0–69.9 kg, 70.0–79.9 kg, and 80.0–90.0 kg.

##### Analysis of Volunteer Baseline Characteristics

Baseline characteristics were summarized using descriptive statistics and compared between groups: continuous variables (age, body weight, laboratory parameters) using the *t*-test (or Mann–Whitney) and categorical variables (sex, medical history, physical exam findings) using the χ^2^ test (or Fisher’s exact), with data presented as counts and percentages.

##### Analysis of PK Parameters

The analysis included the construction of individual and average PK curves for each group based on the individual PK parameter values.

Stata 14 and PKSolver were used for statistical analysis of PK parameters and processing of PK curves.

##### Equivalence Analysis of the PK Parameters of Two Drugs

The equivalence analysis of the PK parameters was performed using a generalized linear regression model (GLM) after logarithmic transformation, with subject body weight (*Wt*, kg) at baseline as a covariate. Thus, the model for estimating the PK parameters was as follows:

(1) $$\ln Y=\beta_{0}+\beta_{1}Group+\beta_{2}Wt,$$

where *Y* is the PK parameter being analyzed, *β*_0_–*β*_2_ are regression coefficients, and *Group* is the volunteer group (1 = trial drug, 0 = comparator drug). Bioequivalence was assessed using 90% CIs for the AUC and Cmax ratios (test/reference) with acceptance limits of 80.00–125.00%.

##### Immunogenicity Analysis

The incidence of ADA formation was compared using the chi-square test (or Fisher’s exact test when expected cell frequencies were <5). The number and percentage of volunteers developing ADAs are presented for each group.

##### Safety Analysis

Adverse event data were coded using the latest MedDRA version at database generation and closure. The incidence of treatment-emergent AEs/serious AEs (SAEs) was calculated by System Organ Class (SOC) and preferred term (PT) per drug, based on the number and percentage of affected volunteers. Summary data on AE severity, causality, and outcomes are also presented. SAEs, AEs leading to withdrawal, and AEs of grade ≥ 3 (NCI CTCAE v.5.0) are reported separately.

### 2.3. Bioanalytical Methods

#### 2.3.1. Serum Sample Preparation and Storage

Blood samples for PK and ADA analysis were collected into clot activator tubes. To prepare serum, the blood was incubated at room temperature for 30 min and then centrifuged at 4000–4500 rpm. Subsequently, 500 µL aliquots of serum were transferred into Eppendorf tubes and stored at −70 °C.

Blood samples for PK analysis were collected at the following time points: 5–15 min before drug administration on Day 1; then at 6 h ± 25 min and 12 h ± 30 min post-dose on Day 1; at 24 h ± 30 min on Day 2; at 48 ± 1 h on Day 3. Thereafter, samples were collected every 24 ± 2 h from Day 4 up to Day 12. Subsequent samples were collected on Day 17 at 384 ± 2 h post-dose, followed by additional collections on Days 21, 28, 35, 49, 63, and 84, with a permissible deviation of ±1 day.

Blood samples for immunogenicity analysis were collected on Day 1 after randomization but strictly before drug administration and subsequently on Day 21, Day 49, and Day 84.

All PK and immunogenicity samples were processed and analyzed by laboratory personnel unaware of the treatment allocation.

#### 2.3.2. PK Assessment

**Figure 1:**
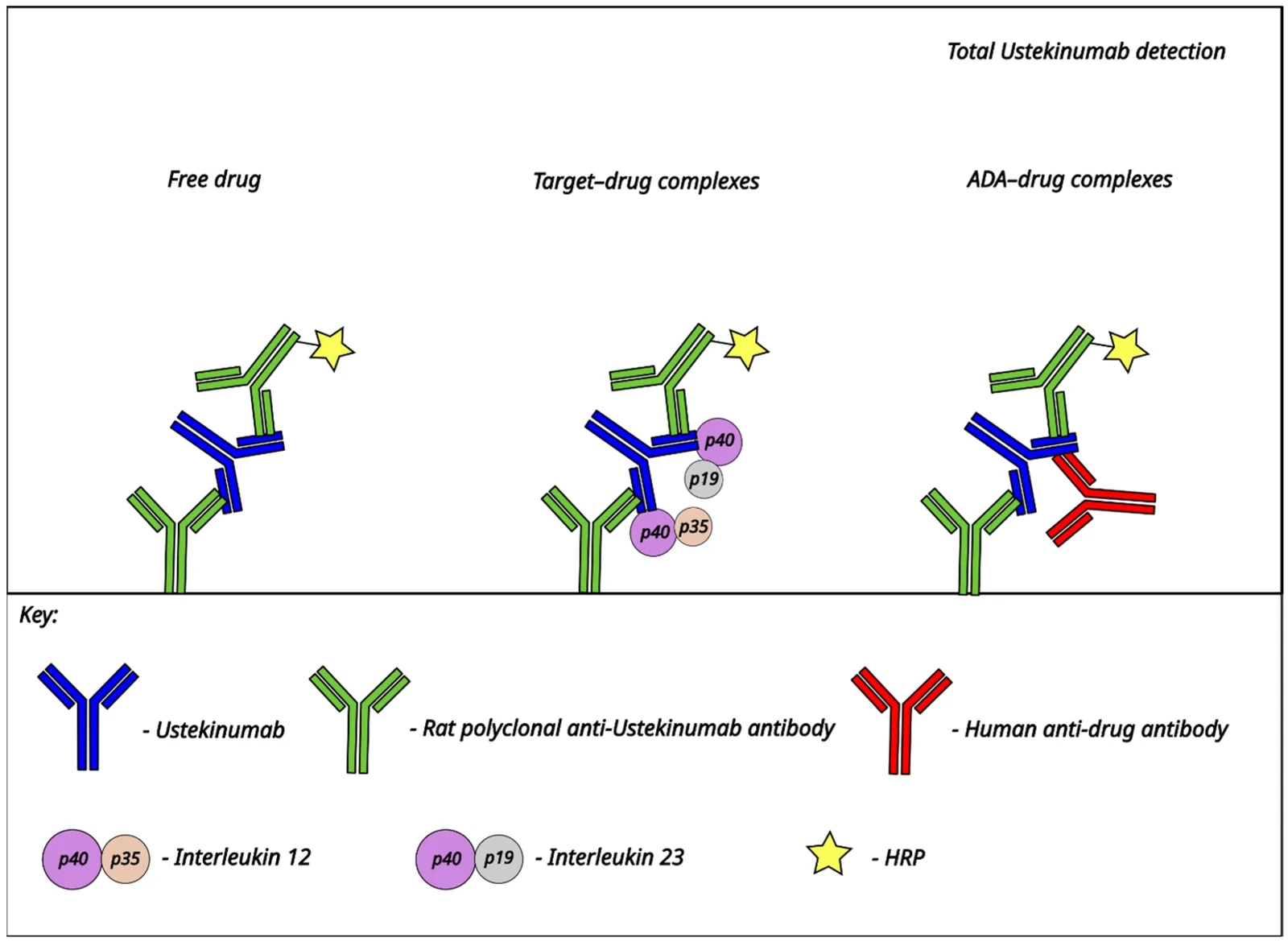
Schematic representation of the ustekinumab concentration assay.

The serum concentrations of ustekinumab were determined using a validated colorimetric enzyme-linked immunosorbent assay (ELISA) (Figure 1). A polyclonal antibody produced in rats against Stelara^®^ was used as the capture antibody. GNR-068 was used as a standard for constructing the calibration curve. The resulting immune complexes were detected using polyclonal rat antibodies to Stelara^®^ conjugated with HRP. After incubation and washing, the chromogenic HRP substrate was added. The reaction was stopped by adding a stop solution, and the absorbance was measured using a plate spectrophotometer at a wavelength of 450 nm. Based on the calibration curve fitted using a 4-parameter function, the concentration of Ustekinumab in the test samples was calculated using Microplate Manager, v. 6.3 (Bio-Rad, Hercules, CA, USA). The lowest quantifiable concentration in a sample for this ELISA was 100 ng/mL [lower limit of quantification (LLOQ) of 1 ng/mL multiplied by the minimum required 1:100 dilution]. Inclusive of all calibration standards, the mean inter-assay accuracy (% relative error) was 93.9 to 108.7%, and the mean inter-assay precision (% coefficient of variation) was 0.9 to 7.0%. Inclusive of three quality control (QC) samples, the mean intra-assay precision ranged from 4.5 to 6.3%. Inclusive of five QC samples, the mean inter-assay accuracy ranged from 101.2 to 107.3%, the mean inter-assay precision ranged from 5.3 to 13.9%, and the total error (mean inter-assay accuracy plus mean inter-assay precision) ranged from 6.5 to 21.2%.

#### 2.3.3. Analysis of Anti-Drug Antibodies

Immunogenicity was assessed using a multi-tiered approach with a screening assay for total ADA, a confirmatory assay, and subsequent titer determination for confirmed positive samples. Positive samples were tested for neutralizing antibodies (NAbs); for those positive, the NAb titer was measured using ELISA. Cut points for the screening and confirmatory assays were established non-parametrically based on non-specific binding observed in serum from ustekinumab-naive subjects.

Human monoclonal antibodies specific to ustekinumab (Cat# HCA210, Bio-Rad, Neuried, Germany) were used as positive controls, and pooled normal human serum served as negative controls (*n* ≥ 30).

**Figure 2:**
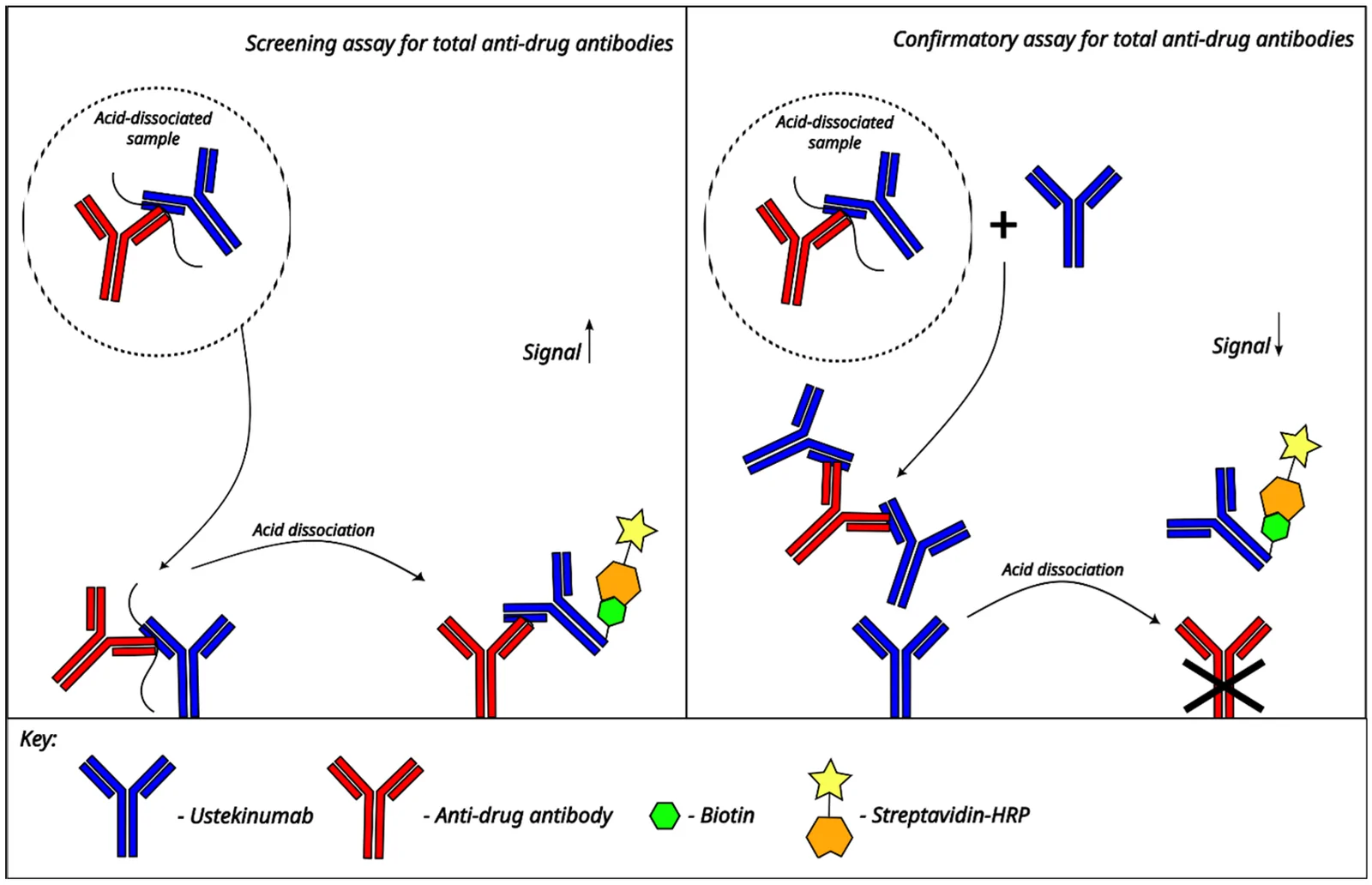
Schematic representation of the screening and confirmatory assays for total ADA. Note: The circle indicates the acid dissociation step of serum samples to disrupt potential complexes formed between ALA and ustekinumab. The wavy line denotes complex disruption. The plus sign (+) in the confirmatory assay indicates the addition of excess unlabeled GNR-068. Long downward arrows indicate the transition from sample preparation to binding with immobilized ustekinumab (and, in the confirmatory assay, with ustekinumab added to the buffer). Long rightward arrows indicate the acid dissociation step and the transition to ALA adsorption onto a new plate and detection using labeled reagents. Short vertical arrows pointing up and down indicate high and low signal, respectively. In the confirmatory assay, the formation of ALA–immobilized drug complexes is reduced due to competition with ustekinumab in the buffer solution, resulting in signal inhibition. The reduction in immobilized complexes is indicated by a cross mark (✕).

The screening assay for total ADA was designed to detect its presence in serum samples (Figure 2, left). GNR-068 was used as the capture reagent. Prior to analysis, serum samples were subjected to acid dissociation to disrupt potential complexes formed between ADA and ustekinumab. ADAs captured on GNR-068 were eluted by acid dissociation and transferred onto another plate for adsorption. Detection was carried out using biotinylated ustekinumab (GNR-068) and HRP-conjugated streptavidin. Tetramethylbenzidine was used for visualization. The reaction was stopped with a stop solution, and optical density was measured at a wavelength of 450 nm using an xMark spectrophotometer (Bio-Rad, Hercules, CA, USA). The total ADA confirmatory assay (Figure 2, right) was similar to the screening assay, except for the inclusion of a competitive inhibition step, in which an excess of unlabeled GNR-068 was added to the buffer for neutralization prior to the binding of ADAs to the immobilized GNR-068. For titer determination, samples were analyzed in serial dilutions using a similar screening assay.

**Figure 3:**
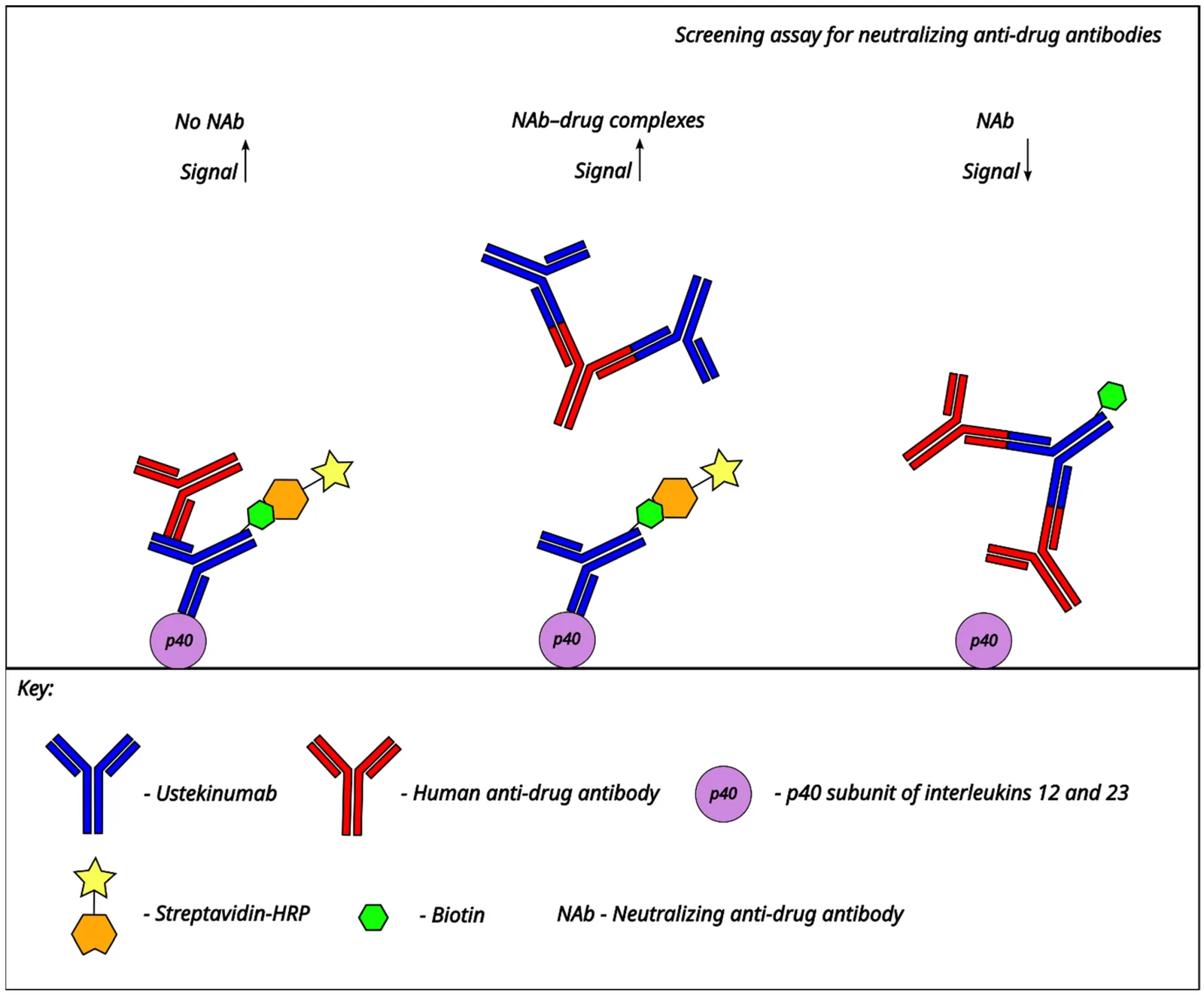
Schematic representation of the ligand-binding competition assay for NAb determination. Note: Short vertical arrows pointing upward and downward indicate high and low signal, respectively. In the absence of NAbs or when NAbs are complexed with the drug present in the sample, they cannot bind to biotinylated ustekinumab, resulting in a high analytical signal. Binding of NAbs present in the sample to biotinylated ustekinumab reduces the number of immobilized complexes, leading to a corresponding decrease in signal.

The screening assay for NAb was based on competitive ligand binding (Figure 3). Serum samples were incubated with biotinylated ustekinumab and then incubated with the immobilized ligand of ustekinumab (the p40 subunit of interleukins 12 and 23), followed by detection using HRP-conjugated streptavidin. Visualization and signal readout were performed similarly to the total ADA assay methodology. Titers were determined in serial dilutions using an assay analogous to the screening assay.

Data obtained using Microplate Manager, v. 6.3 (Bio-Rad, Hercules, CA, USA) were processed and analyzed in Microsoft Excel 2016 (Microsoft Corp., Redmond, WA, USA).

Total and neutralizing ADAs were evaluated using validated assays. The sensitivity of the total ADA assay was 112 ng/mL at a minimum required dilution (MRD) of 1:10 and 405 ng/mL for the NAb assay at an MRD of 1:5. For the total ADA screening assay, the intra-assay precision for positive control samples at concentrations of 10, 1, and 0.25 µg/mL was 11.6%, 14.9%, and 14.0%, respectively, while the inter-assay precision was 6.3%, 21.5%, and 17.5%. For the NAb screening assay, the intra-assay precision for positive control samples at concentrations of 5, 1, and 0.5 µg/mL was 0.5%, 1.2%, and 6.9%, respectively, and the inter-assay precision was 0.4%, 3.4%, and 21.0%.

## 3. Results

### 3.1. Trial Participant Characteristics

**Figure 4:**
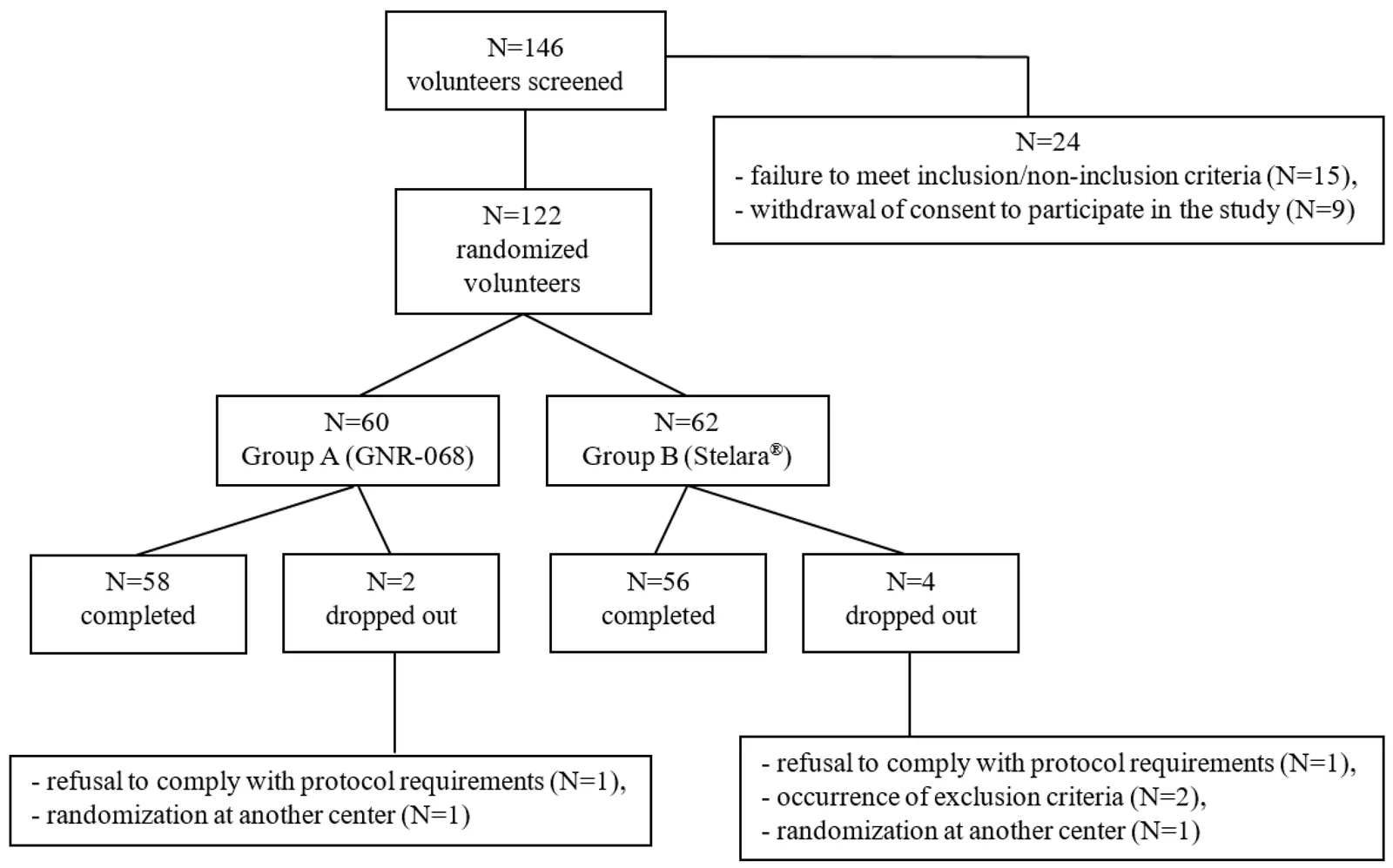
Distribution of volunteers.

A total of 146 volunteers were screened, and 122 were randomized (60 to the GNR-068 group and 62 to the Stelara^®^ group) (Figure 4). It was subsequently discovered that two volunteers had undergone re-randomization and received a second injection: although initially assigned to different groups, both were allocated to the Stelara^®^ group upon re-randomization. Only data obtained from these volunteers up to the second ustekinumab administration were included in the analysis. As a result, data from 120 volunteers were included in the statistical analysis and constituted the full analysis set (FAS), as well as the PK and safety populations. Safety data obtained from the two volunteers after the second ustekinumab administration were analyzed separately. Of the 120 volunteers, 116 (96.7%) completed the study without major protocol deviations, and 4 (3.3%) discontinued prematurely, including the two volunteers who were re-randomized. 

The trial included healthy volunteers aged 19 to 44 years, with an average age of 32.5 ± 6.3 years in Group A (GNR-068) and 31.9 ± 7.5 years in Group B (Stelara^®^). No statistically significant differences in age, height, body weight, and body mass index were found between the groups (Table 1; *p* > 0.05). All volunteers were Caucasian males; one volunteer was Asian.

### 3.2. Pharmacokinetics

**Figure 5:**
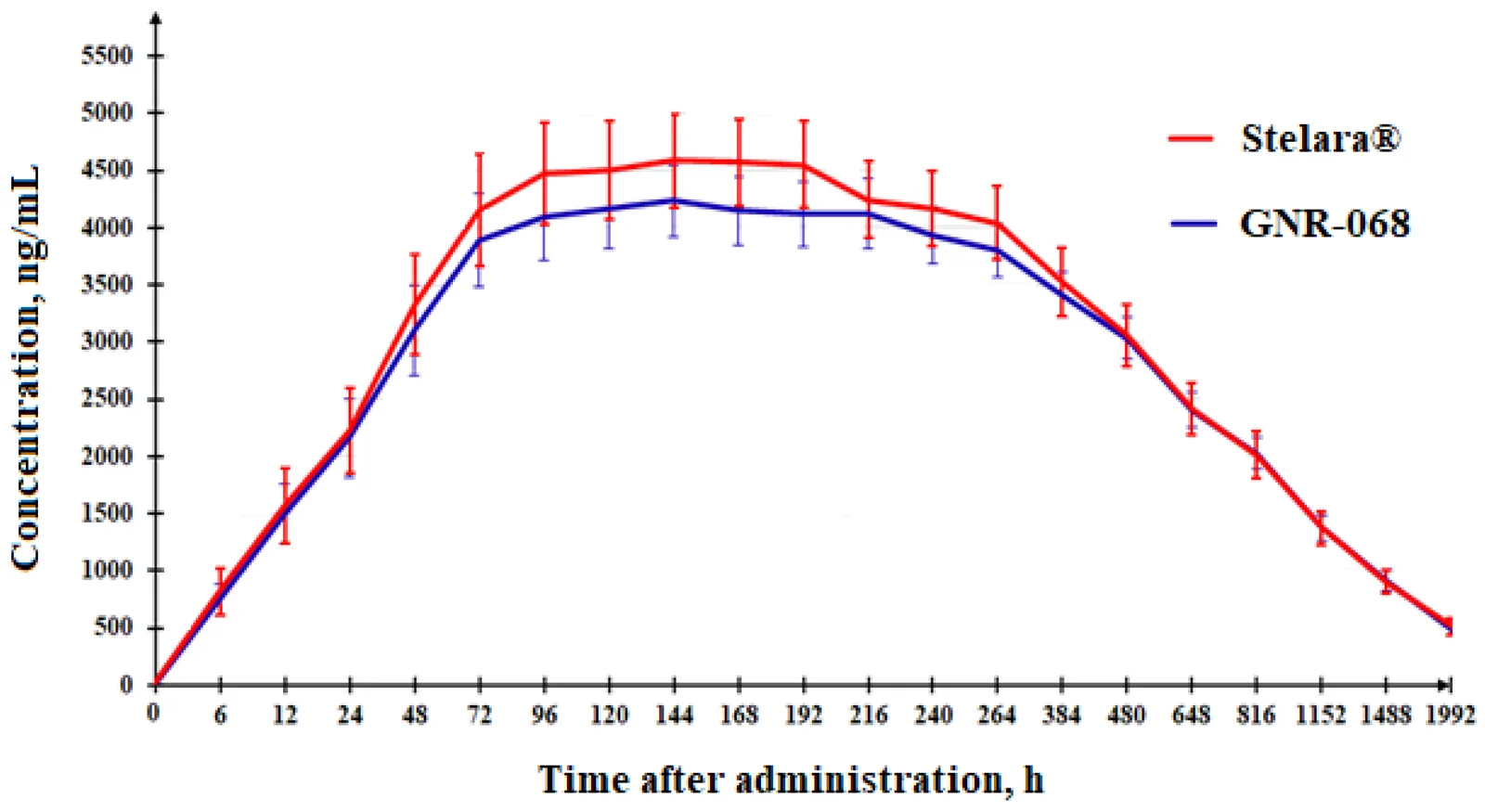
Time course changes in ustekinumab serum concentration. Red line: Stelara^®^ group; blue line: GNR-068. Error bars show the 95% confidence interval (CI).

The PK analysis population comprised 120 healthy volunteers who had evaluable PK data for at least one parameter. Following a single subcutaneous injection of ustekinumab, the maximum mean serum concentration was observed at 144 ± 2 h after administration (4231.58 ± 1244.90 ng/mL for GNR-068 and 4582.58 ± 1606.07 for Stelara^®^, *p* = 0.184), followed by a decrease in mean concentration to the last time point at 1992 ± 24 h after administration (to 488.42 ± 254.09 ng/mL for GNR-068 and 512.36 ± 288.58 for Stelara^®^, *p* = 0.636). The intergroup comparison of GNR-068 and Stelara^®^ concentrations at different time points (Table 2, Figure 5), as well as of the key PK parameters (Table 3) using a *t*-test, revealed no statistically significant differences (*p* > 0.05).

### 3.3. Analysis of the Equivalence of PK Parameters of Two Drugs

Analysis of the primary pharmacokinetic parameters of GNR-068 and Stelara^®^, performed using a log-transformed generalized linear model with body weight as a covariate, confirmed the equivalence of the two products. The 90% confidence intervals for the ratios of AUC_0–t_, AUC_0–∞_, and Cmax fell entirely within the prespecified equivalence range of 80.00–125.00% (Table 4).

### 3.4. Safety

The trial reported 148 adverse events (AEs) in 57 volunteers (47.5%): 24 volunteers (40.0%) in Group A and 33 volunteers (55.0%) in Group B. No statistically significant differences were found between the groups.

**Figure 6:**
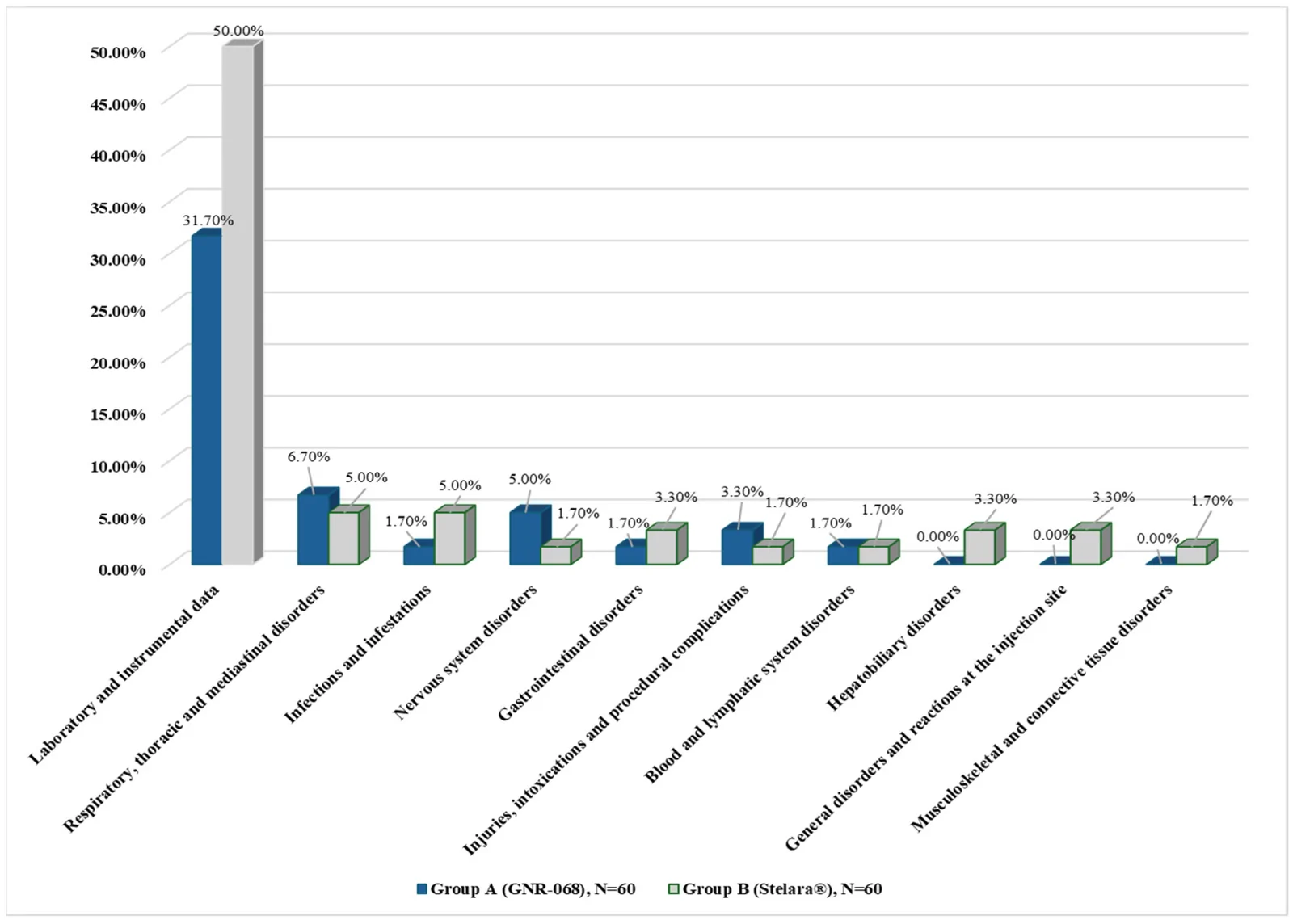
Proportion of volunteers with treatment-emergent adverse events by preferred term (PT, MedDRA).

The 148 AEs recorded during the trial involved 10 System Organ Classes (SOCs), including gastrointestinal disorders, infections and infestations, nervous system disorders, and others (see Figure 6).

In 25 volunteers, 48 ARs of probable and possible association were recorded: 12 volunteers (20.0%) in Group A and 13 volunteers (21.7%) in Group B. Notably, 3 ARs in the GNR-068 group were probable associations (two gastrointestinal disorders and one infection and infestation), and 45 ARs were possibly associated (20 ARs in the GNR-068 group and 25 ARs in the Stelara^®^ group).

The vast majority of AEs recorded in the study (136 episodes) were mild in severity. One SAE was reported in Group I (SOC “Injuries, intoxications, and procedural complications”; PT “clavicle fracture”), which was not related to the study drug. No deaths occurred in this study.

### 3.5. Immunogenicity

A statistically significant difference in the rate of total ADA formation was observed: ADA development was noted in 20 (33.3%) volunteers in Group A compared with 46 (76.7%) in the comparator group (*p* < 0.001). Of these, 2 volunteers (3.3%) in Group A and 8 volunteers (13.3%) in Group B had detectable anti-drug antibodies at Visit 2 before drug administration (Table 5).

In 105 out of 144 ADA detection cases (72.9%), the titer was low (below 1:100). In the majority of volunteers with a detected ADA response, the titer remained at a similar level throughout the trial, including in those with ADA detected before drug administration (Table 6).

Neutralizing antibody formation was observed in 3 volunteers (5.0%) in Group A and in 2 volunteers (3.3%) in Group B (*p* = 1.000; Table 7).

## 4. Discussion

The primary objective of this study was to evaluate the pharmacokinetic equivalence as well as to compare the immunogenicity and safety profiles of GNR-068 and Stelara^®^. The study was conducted in healthy male volunteers following a single subcutaneous injection of 45 mg.

The analytical methods used for drug concentration determination and immunogenicity assessment were validated and complied with current regulatory requirements [22,23]. The ustekinumab quantification assay was straightforward, provided high throughput, and demonstrated a sensitivity of 100 ng/mL. A distinctive feature of the anti-ustekinumab total ADA assay was the inclusion of an elution step, during which ADAs were released from complexes with the immobilized drug, followed by adsorption and detection on a separate plate. This design ensured high sensitivity (112 ng/mL). To mitigate drug interference, serum samples underwent acid dissociation prior to analysis. The neutralizing activity of detected ADAs was evaluated by measuring the inhibition of ustekinumab binding to its target.

Analysis of the primary pharmacokinetic parameters (AUC_0–t_, AUC_0–∞_, and Cmax) showed that the 90% confidence intervals for the geometric mean ratios were entirely contained within the equivalence range of 80.00–125.00%. Following a single 45 mg subcutaneous injection, ustekinumab was slowly absorbed into the systemic circulation, with a median time to maximum concentration (Tmax) of approximately one week. Individual Tmax values ranged from 3.0 to 16.0 days in the GNR-068 group and from 3 to 20 days in the Stelara^®^ group, indicating high interindividual variability in absorption. After reaching Cmax, mean serum ustekinumab concentrations declined monoexponentially until the end of the study, consistent with the one-compartment open model previously described for ustekinumab in population pharmacokinetic analyses of patients with psoriasis or psoriatic arthritis [24,25].

Ustekinumab was eliminated from the circulation with a mean half-life (T1/2) of approximately 24 days, which aligns with previously reported values in patients with psoriasis (21.6 days) or psoriatic arthritis (21.2 days) [24,25] and is consistent with the half-life of other fully human therapeutic IgG1 monoclonal antibodies [26,27]. Body weight represents a significant covariate affecting the clearance of ustekinumab, as is the case for many other IgG monoclonal antibodies [26,27].

Single 45 mg subcutaneous injections of ustekinumab were generally well tolerated in healthy volunteers. The incidence of total ADA was lower in the GNR-068 group compared with the reference product group.

The investigational product GNR-068, like Stelara^®^, is manufactured using the murine myeloma Sp2/0 cell line. According to published data, many other ustekinumab biosimilars are produced using Chinese hamster ovary (CHO) cell lines [28]. Clinical studies of several such biosimilars have shown lower ADA incidence in the investigational product groups compared with the reference product groups [29,30,31]. For example, in the clinical trial of the biosimilar Wezlana (Amgen), the total ADA incidence in the biosimilar group (15.4%) was lower than in the comparator groups (38.0% and 36.3% for US-sourced and EU-sourced ustekinumab, respectively). A similar trend was observed for NAb: 2.6% in the Wezlana group versus 12.7% and 7.5% in the comparator groups [29].

Differences in immunogenicity profiles may be attributed to differences in glycosylation patterns [32,33,34]. Both Sp2/0 and CHO cell lines produce non-human glycan structures, including galactose α1,3-galactose (α-Gal) and N-glycolylneuraminic acid (NGNA). However, CHO cells are reported to provide a glycosylation profile that is closer to that of humans [35]. Interestingly, lower immunogenicity was also observed for the biosimilar Selarsdi (Alvotech), which is produced in the Sp2/0 cell line [36].

We hypothesize that the statistically significant difference in total ADA incidence between the groups in our study may be explained by the presence of pre-existing antibodies against NGNA. The prevalence and magnitude of this antibody response may have been unevenly distributed between the groups because this factor was not assessed or accounted for at randomization. This hypothesis is supported by the detection of pre-dose antibodies in healthy volunteers with no prior exposure to the drug. Moreover, the observed differences in ADA responses may have been amplified by differences in the glycosylation profiles of the products, even though they are produced using the same cell line. It is important to emphasize that the observed difference in ADA incidence did not lead to any differences in pharmacokinetic parameters between the groups. Furthermore, the presence of ADAs was not associated with hypersensitivity reactions, severe anaphylaxis, or injection-site reactions in either treatment group.

Based on the results obtained, it can be concluded that GNR-068 and the reference product Stelara^®^ are well tolerated, exhibit comparable safety profiles, and are pharmacokinetically equivalent following a single subcutaneous administration to healthy volunteers. However, this study has several limitations. The results obtained in healthy volunteers cannot be directly extrapolated to patients with psoriasis. The single-dose design, small sample size, and short follow-up period were appropriate for assessing pharmacokinetic equivalence but may have been insufficient to detect rare or delayed adverse reactions or to accurately determine the incidence of ADA formation. Therefore, the immunogenicity of GNR-068 and Stelara^®^ will be further evaluated in a subsequent comparative efficacy study in patients with moderate-to-severe plaque psoriasis. Additionally, the open-label design represents a potential source of bias. However, to mitigate this risk, blinding was implemented at the analytical stage: all PK and immunogenicity samples were processed and analyzed by laboratory personnel unaware of the treatment allocation. Nevertheless, the lack of blinding for investigators and subjects remains a limitation that should be considered when interpreting safety and tolerability outcomes.

## 5. Conclusions

The two formulations showed no clinically significant safety differences in healthy volunteers after a single 45 mg subcutaneous dose. The trial demonstrated pharmacokinetic equivalence between GNR-068 and Stelara^®^, with the 90% CI for the geometric mean ratios of Cmax, AUC_0–t_, and AUC_0–∞_ falling completely within the equivalence recognition range of 80.00–125.00%. Statistically significant differences in total ADA levels were found between groups (higher in the comparator group), but no differences in the frequency of neutralizing antibodies were detected.

## Figures and Tables

**Table 1:** Demographic characteristics of volunteers (FAS population).

Indicator	Parameter	Group A (GNR-068)	Group B (Stelara^®^)	Total	*p*
Age, years	N	60	60	120	0.618
	M	32.5	31.9	32.2	
	SD	6.3	7.5	6.9	
	95% CI	(30.9; 34.2)	(30.0; 33.8)	(31.0; 33.5)	
	Min	20	19	19	
	Max	44	44	44	
	Me	33	32	32	
	IQR	10.5	14	11.5	
Height, cm	N	60	60	120	0.706
	M	178.2	177.7	178.0	
	SD	6.8	6.2	6.5	
	95% CI	(176.4; 180.0)	(176.1; 179.3)	(176.8; 179.1)	
	Min	160	162	160	
	Max	196	193	196	
	Me	180	178	178	
	IQR	10.5	8	8.5	
Weight, kg	N	60	60	120	0.821
	M	75.73	76.10	75.91	
	SD	8.98	9.02	8.97	
	95% CI	(73.41; 78.05)	(73.77; 78.43)	(74.29; 77.53)	
	Min	60.6	60.5	60.5	
	Max	89.9	89.8	89.9	
	Me	75.55	75	75.3	
	IQR	15.7	17.6	16.45	
Body mass index, kg/m^2^	N	60	60	120	0.574
	M	23.83	24.07	23.95	
	SD	2.28	2.36	2.31	
	95% CI	(23.24; 24.42)	(23.46; 24.68)	(23.53; 24.37)	
	Min	19	19.6	19	
	Max	27.5	29.2	29.2	
	Me	23.75	23.8	23.8	
	IQR	3.35	3.3	3.3	

Note: N—number of subjects; M—mean; SD—standard deviation; 95% CI—95% confidence interval; Min—minimum; Max—maximum; Me—median; IQR—interquartile range; *p*—*p*-value.

**Table 2:** Descriptive statistics of the concentration of the trial drug GNR-068 and the comparator drug Stelara^®^ (ng/mL) over 1992 h (PK population).

**Parameter**	**Before Injection**		**6 h**		**12 h**		**24 h**		**48 h**		**72 h**		**96 h**	
	**GNR-068**	**Stelara^®^**	**GNR-068**	**Stelara^®^**	**GNR-068**	**Stelara^®^**	**GNR-068**	**Stelara^®^**	**GNR-068**	**Stelara^®^**	**GNR-068**	**Stelara^®^**	**GNR-068**	**Stelara^®^**
N	60	60	60	60	60	60	60	60	60	60	60	59	60	60
M	3.66	19.15	745.82	817.95	1494.38	1567.34	2158.39	2225.51	3103.11	3323.80	3889.14	4154.24	4085.75	4471.67
SD	16.46	92.93	534.59	816.67	1053.69	1298.92	1337.38	1471.60	1550.81	1740.89	1598.61	1926.07	1471.09	1759.63
95% CI	(0.00; 7.91)	(0.00; 43.16)	(607.72; 883.92)	(606.98; 1028.92)	(1222.19; 1766.58)	(1231.80; 1902.89)	(1812.91; 2503.87)	(1845.35; 2605.66)	(2702.50; 3503.73)	(2874.08; 3773.52)	(3476.18; 4302.10)	(3652.30; 4656.18)	(3705.72; 4465.77)	(4017.11; 4926.23)
Min	0.0	0.0	0.0	0.0	172.9	101.1	328.0	167.1	441.9	293.8	959.3	426.0	1071.4	558.3
Max	111.6	663.4	2769.6	4340.2	5076.6	7201.6	6607.5	7229.1	7637.9	7904.0	8737.4	8681.8	8812.4	8392.7
Me	0.0	0.0	763.5	612.4	1325.4	1341.2	1916.1	2059.0	2792.1	3311.3	3673.0	3796.3	3917.3	4124.2
IQR	0.0	0.0	658.3	821.8	1616.6	1499.6	1793.1	2082.3	2483.7	2430.9	2456.4	2720.6	2140.0	2333.6
CV	449.6%	485.2%	71.7%	99.8%	70.5%	82.9%	62.0%	66.1%	50.0%	52.4%	41.1%	46.4%	36.0%	39.4%
*p* (*t*-test)	0.206		0.568		0.736		0.794		0.465		0.415		0.195	
**Parameter**	**120 h**		**144 h**		**168 h**		**192 h**		**216 h**		**240 h**		**264 h**	
	**GNR-068**	**Stelara^®^**	**GNR-068**	**Stelara^®^**	**GNR-068**	**Stelara^®^**	**GNR-068**	**Stelara^®^**	**GNR-068**	**Stelara^®^**	**GNR-068**	**Stelara^®^**	**GNR-068**	**Stelara^®^**
N	60	60	60	60	60	59	59	59	60	58	59	60	59	60
M	4164.24	4497.93	4231.58	4582.58	4149.56	4569.09	4116.79	4551.17	4120.73	4241.73	3936.66	4161.18	3801.42	4039.43
SD	1390.28	1692.69	1244.90	1606.07	1177.09	1496.05	1128.31	1485.12	1208.24	1309.12	980.87	1301.51	909.86	1270.05
95% CI	(3805.10; 4523.39)	(4060.67; 4935.20)	(3909.99; 4553.17)	(4167.69; 4997.47)	(3845.48; 4453.63)	(4179.22; 4958.97)	(3822.75; 4410.83)	(4164.15; 4938.20)	(3808.61; 4432.85)	(3897.51; 4585.94)	(3681.05; 4192.28)	(3824.96; 4497.40)	(3564.31; 4038.53)	(3711.34; 4367.51)
Min	1188.6	638.5	1409.7	702.5	1481.6	814.5	1662.2	967.4	1569.2	867.9	1428.5	976.5	1699.0	956.9
Max	8139.6	8431.4	7807.0	8979.9	7160.6	8225.3	6935.1	9205.4	7572.1	7987.3	5932.3	7791.9	5951.4	7215.8
Me	3989.9	4248.8	4095.2	4505.0	4030.8	4384.9	3942.8	4420.8	3974.2	4241.1	3892.0	4148.4	3780.3	4013.3
IQR	1856.4	2277.1	1824.8	2091.2	1687.0	1962.4	1559.2	1913.4	1390.0	1513.7	1444.5	1793.8	1365.2	1529.1
CV	33.4%	37.6%	29.4%	35.0%	28.4%	32.7%	27.4%	32.6%	29.3%	30.9%	24.9%	31.3%	23.9%	31.4%
*p* (*t*-test)	0.240		0.184		0.092		0.076		0.603		0.291		0.243	
**Parameter**	**384 h**		**480 h**		**648 h**		**816 h**		**1152 h**		**1488 h**		**1992 h**	
	**GNR-068**	**Stelara^®^**	**GNR-068**	**Stelara^®^**	**GNR-068**	**Stelara^®^**	**GNR-068**	**Stelara^®^**	**GNR-068**	**Stelara^®^**	**GNR-068**	**Stelara^®^**	**GNR-068**	**Stelara^®^**
N	59	60	58	60	58	59	59	60	57	60	55	59	58	58
M	3413.33	3521.93	3033.35	3056.82	2402.52	2413.52	2025.02	2009.35	1370.77	1373.67	917.14	899.44	488.42	512.36
SD	777.61	1165.15	731.91	1084.20	599.15	871.39	531.09	791.60	408.89	568.97	342.63	402.11	254.09	288.58
95% CI	(3210.69; 3615.98)	(3220.94; 3822.92)	(2840.90; 3225.79)	(2776.74; 3336.90)	(2244.99; 2560.06)	(2186.43; 2640.60)	(1886.61; 2163.42)	(1804.86; 2213.85)	(1262.27; 1479.26)	(1226.69; 1520.66)	(824.51; 1009.76)	(794.65; 1004.23)	(421.61; 555.23)	(436.48; 588.23)
Min	1587.3	640.3	1391.0	366.3	1161.7	280.6	991.1	144.0	540.3	59.1	0.0	0.0	0.0	0.0
Max	5330.1	6223.0	4716.7	5732.9	3733.7	4633.2	3133.0	3619.6	2476.6	2630.4	2093.3	1702.9	1224.3	1242.8
Me	3325.9	3477.2	3031.0	3141.4	2369.4	2491.4	1943.0	2080.7	1325.3	1456.8	934.6	952.4	495.7	515.9
IQR	1138.1	1215.5	904.3	1126.7	778.6	798.8	833.9	1070.1	513.7	724.9	354.9	532.4	258.4	363.6
CV	22.8%	33.1%	24.1%	35.5%	24.9%	36.1%	26.2%	39.4%	29.8%	41.4%	37.4%	44.7%	52.0%	56.3%
*p* (*t*-test)	0.552		0.891		0.937		0.900		0.975		0.802		0.636	

Note: N—number of subjects; M—mean; SD—standard deviation; 95% CI—95% confidence interval; Min—minimum; Max—maximum; Me—median; IQR—interquartile range; *p*—*p*-value.

**Table 3:** Descriptive statistics of the main PK parameters (PK population) adjusted for body weight.

**Parameters**	**Tmax, h**		**Cmax, ng/mL**		**AUC_0_** **_–_** **_∞_** **, h·mkg/mL**		**AUC_0_** **_–_** **_t_** **, h·mkg/mL**	
	**GNR-068**	**Stelara^®^**	**GNR-068**	**Stelara^®^**	**GNR-068**	**Stelara^®^**	**GNR-068**	**Stelara^®^**
N	60	60	60	60	60	60	60	60
M	157.22	159.38	4653.69	4956.46	4289.31	4419.39	3813.03	3945.69
SD	64.31	67.33	1419.22	1686.88	1202.85	1588.49	1019.01	1363.94
95% CI	(140.61; 173.83)	(141.99; 176.78)	(4287.06; 5020.31)	(4520.69; 5392.23)	(3978.58; 4600.03)	(4009.04; 4829.74)	(3549.79; 4076.27)	(3593.34; 4298.03)
Min	70.4	71.1	1699.0	1004.4	1885.3	853.8	1623.6	833.0
Max	385.1	480.6	8812.4	9205.4	7419.5	8520.0	5920.1	7554.6
Me	144.4	145.0	4429.6	4723.5	4026.6	4689.0	3670.5	4084.1
IQR	108.0	72.2	1993.3	2210.4	1580.8	1496.7	1433.2	1640.0
CV, %	40.9%	42.2%	30.5%	34.0%	28.0%	35.9%	26.7%	34.6%
*p* (*t*-test)	0.858		0.290		0.614		0.547	
**Parameters**	**Kel, h^−1^**		**T1/2, h**		**Vd, L**		**CL, L/h**	
	**GNR-068**	**Stelara^®^**	**GNR-068**	**Stelara^®^**	**GNR-068**	**Stelara^®^**	**GNR-068**	**Stelara^®^**
N	60	60	60	60	60	60	60	60
M	0.0013	0.0013	588.24	571.97	0.0093	0.0095	0.0114	0.0126
SD	0.0003	0.0005	145.42	151.67	0.0027	0.0046	0.0036	0.0084
95% CI	(0.0012; 0.0013)	(0.0012; 0.0015)	(550.68; 625.81)	(532.79; 611.15)	(0.0086; 0.0100)	(0.0083; 0.0107)	(0.0105; 0.0123)	(0.0105; 0.0148)
Min	0.0006	0.0008	305.5	203.5	0.0053	0.0048	0.0061	0.0053
Max	0.0023	0.0034	1164.3	860.2	0.0202	0.0279	0.0239	0.0527
Me	0.0012	0.0012	580.4	567.6	0.0087	0.0083	0.0112	0.0096
IQR	0.0003	0.0004	130.4	190.1	0.0029	0.0036	0.0039	0.0034
CV, %	25.7%	39.2%	24.7%	26.5%	28.8%	48.3%	31.6%	66.7%
*p* (*t*-test)	0.300		0.550		0.820		0.298	

Note: Tmax, h,—time to reach maximum serum concentration; Cmax, ng/mL—maximum serum concentration; AUC_0–∞_, h·mkg/mL—area under the curve extrapolated to infinity; AUC_0–t_, h·mkg/mL—area under the curve from time 0 to last measurable time point; Kel, h^−1^—elimination rate constant; T1/2, h—elimination half-life; Vd, L—volume of distribution; CL, L/h—total body clearance; N—number of subjects; M—mean; SD—standard deviation; 95% CI—95% confidence interval; Min—minimum; Max—maximum; Me—median; IQR—interquartile range; *p*—*p*-value.

**Table 4:** Results of the comparison of various PK parameters between the trial drug (GNR-068) and the reference drug (Stelara^®^).

Parameters	Ratio of Parameters for Two Drugs (T/R)			*p*
	Point Estimate	90% CI		
Cmax	94.94%	86.75%	103.91%	0.344
AUC_0–∞_	100.93%	90.80%	112.19%	0.885
AUC_0–t_	99.77%	90.38%	110.13%	0.969

**Table 5:** Frequency of detection of total anti-drug antibodies.

Anti-Drug Antibodies	Visit 2		Visit 15		Visit 18		Visit 20		During the Trial	
	GNR-068	Stelara^®^	GNR-068	Stelara^®^	GNR-068	Stelara^®^	GNR-068	Stelara^®^	GNR-068	Stelara^®^
Discovered	2/60 (3.3%)	8/60 (13.3%)	11/58 (19.0%)	38/60 (63.3%)	12/58 (20.7%)	28/60 (46.7%)	14/58 (24.1%)	28/58 (48.2%)	20/60 (33.3%)	46/60 (76.7%)
*p*	0.095		<0.001		0.003		0.007		<0.001	

**Table 6:** Descriptive statistics of data on total antibody titers at different visits.

Parameters	Visit 2		Visit 15		Visit 18		Visit 20	
	GNR-068	Stelara^®^	GNR-068	Stelara^®^	GNR-068	Stelara^®^	GNR-068	Stelara^®^
N	2	8	11	38	12	28	14	28
M	122.5	716.3	85.5	426.2	102.7	359.4	391.4	368.0
SD	79.9	1706.6	101.2	922.9	226.5	895.1	684.7	924.2
gMean	108.7	75.3	52.9	105.0	43.5	69.4	80.3	79.4
95% CI for gMean	(0.2; 61,525.6)	(11.9; 475.1)	(27.3; 102.4)	(61.9; 178.1)	(22.5; 84.2)	(37.2; 129.6)	(28.5; 225.7)	(43.3; 145.7)
Min	66	12	14	17	17	16	10	11
Max	179	4921	349	4507	819	4421	1726	4701
Me	122.5	38.5	49	56	36	42.5	46	49
IQR	113	336	76	423	22.5	138	130	134
*p*	0.651		0.231		0.337		0.934	

**Table 7:** Frequency of detection of neutralizing anti-drug antibodies (safety population).

Anti-Drug Antibodies	Visit 2		Visit 15		Visit 18		Visit 20		During the Trial	
	GNR-068	Stelara^®^	GNR-068	Stelara^®^	GNR-068	Stelara^®^	GNR-068	Stelara^®^	GNR-068	Stelara^®^
Discovered	0/60 (0.0%)	0/60 (0.0%)	1/58 (1.7%)	2/60 (3.3%)	1/58 (1.7%)	0/60 (0.0%)	2/58 (3.5%)	0/58 (0.0%)	3/60 (5.0%)	2/60 (3.3%)
*p*	-		1.000		0.492		0.496		1.000	

## Data Availability

The original contributions presented in this study are included in the article. Further inquiries can be directed to the corresponding author.

## Abbreviations

The following abbreviations are used in this manuscript:

RP	Reference product
QC	Quality control
PK	Pharmacokinetics
NAb	Neutralizing antibodies
CHO	Chinese hamster ovary
ADA	Anti-drug antibodies
µL	Microliter
ng/mL	Nanograms per milliliter
